# Supplementary material for: Causal Relationship between Aging and Anorexia Nervosa: A White-Matter-Microstructure-Mediated Mendelian Randomization Analysis
Source: Biomedicines. 2024 Aug 16;12(8):1874. doi: 10.3390/biomedicines12081874 (PMC11351342; doi:10.3390/biomedicines12081874)
Supplement: Supplementary file 1 [file biomedicines-12-01874-s001.zip › Supplementary Figure/Figure S4/Singe SNP plot of MR analysis for TL to ubm-b-1522.pdf]

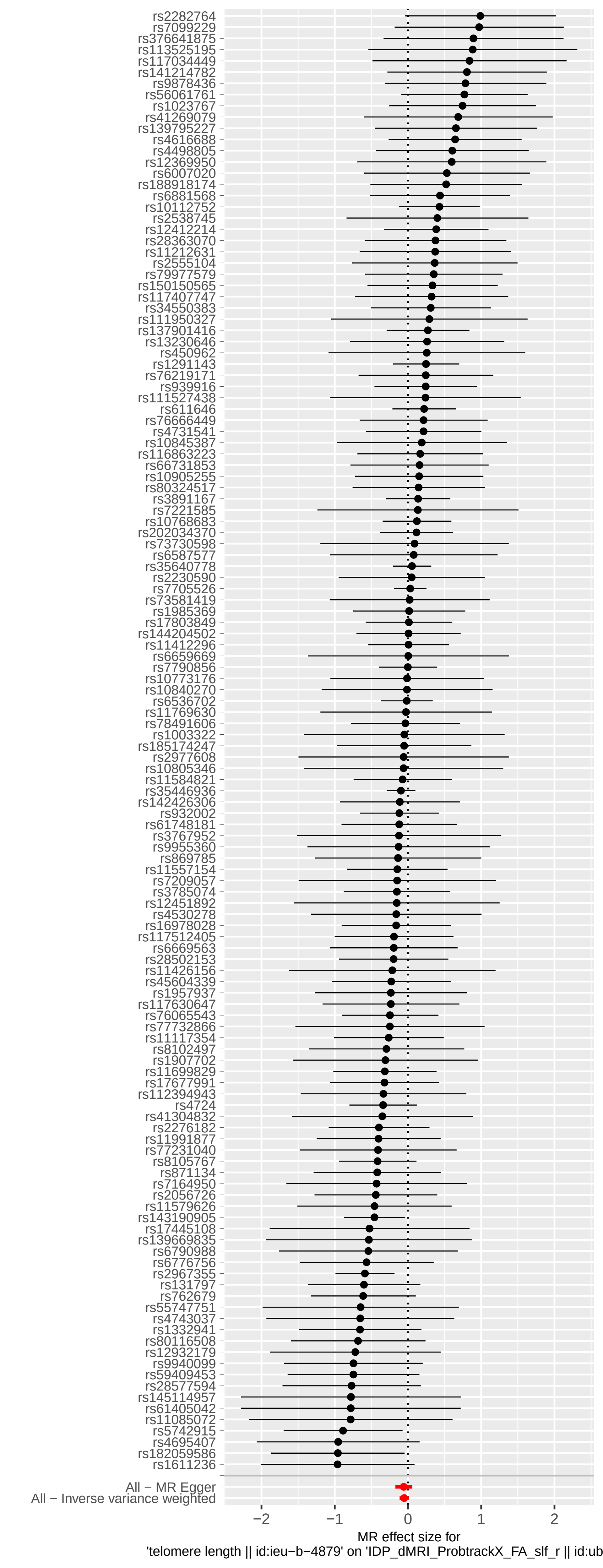
rs2282764
rs7099229
rs376641875
rs113525195
rs117034449
rs141214782
rs9878436
rs56061761
rs1023767
rs41269079
rs139795227
rs4616688
rs4498805
rs12369950
rs6007020
rs188918174
rs6881568
rs10112752
rs2538745
rs12412214
rs28363070
rs11212631
rs2555104
rs79977579
rs150150565
rs117407747
rs34550383
rs111950327
rs137901416
rs13230646
rs450962
rs1291143
rs76219171
rs939916
rs111527438
rs611646
rs76666449
rs4731541
rs10845387
rs116863223
rs66731853
rs10905255
rs80324517
rs3891167
rs7221585
rs10768683
rs202034370
rs73730598
rs6587577
rs35640778
rs2230590
rs7705526
rs73581419
rs1985369
rs17803849
rs144204502
rs11412296
rs6659669
rs7790856
rs10773176
rs10840270
rs6536702
rs11769630
rs78491606
rs1003322
rs185174247
rs2977608
rs10805346
rs11584821
rs35446936
rs142426306
rs932002
rs61748181
rs3767952
rs9955360
rs869785
rs11557154
rs7209057
rs3785074
rs12451892
rs4530278
rs16978028
rs117512405
rs6669563
rs28502153
rs11426156
rs45604339
rs1957937
rs117630647
rs76065543
rs77732866
rs11117354
rs8102497
rs1907702
rs11699829
rs17677991
rs112394943
rs4724
rs41304832
rs2276182
rs11991877
rs77231040
rs8105767
rs871134
rs7164950
rs2056726
rs11579626
rs143190905
rs17445108
rs139669835
rs6790988
rs6776756
rs2967355
rs131797
rs762679
rs55747751
rs4743037
rs1332941
rs80116508
rs12932179
rs9940099
rs59409453
rs28577594
rs145114957
rs61405042
rs11085072
rs5742915
rs4695407
rs182059586
rs1611236
All − MR Egger
All − Inverse variance weighted
−2
−1
0
1
2
MR effect size for
'telomere length || id:ieu−b−4879' on 'IDP_dMRI_ProbtrackX_FA_slf_r || id:ub
